# Human FOXP3^+^ T regulatory cell heterogeneity

**DOI:** 10.1002/cti2.1005

**Published:** 2018-01-30

**Authors:** Audrey Mohr, Rajneesh Malhotra, Gaell Mayer, Guy Gorochov, Makoto Miyara

**Affiliations:** ^1^ Sorbonne Université Inserm Centre d'immunologie et des maladies infectieuses‐Paris (Cimi‐Paris) AP‐HP Hôpital Pitié‐Salpêtrière Paris France; ^2^ Immunity department RIA IMED Biotech Unit AstraZeneca Gothenburg Mölndal Sweden; ^3^ Biometrics & Information Sciences Respiratory, Inflammation, Autoimmunity & Neurosciences Global Medicine Development, AstraZeneca Mölndal Sweden; ^4^ Département d'Immunologie AP‐HP, Groupement Hospitalier Pitié‐Salpêtrière Paris France

**Keywords:** epigenetics, immunotherapy, regulatory T cells

## Abstract

FOXP3‐expressing CD4^+^ T regulatory (Treg) cells are instrumental for the maintenance of self‐tolerance. They are also involved in the prevention of allergy, allograft rejection, foetal rejection during pregnancy and of exaggerated immune response towards commensal pathogens in mucosal tissues. They can also prevent immune responses against tumors and promote tumor progression. FOXP3‐expressing Treg cells are not a homogenous population. The different subsets of Treg cells can have different functions or roles in the maintenance of immune homeostasis and can therefore be differentially targeted in the management of autoimmune diseases or in cancer. We discuss here how Treg cell subsets can be differentiated phenotypically, functionally and developmentally in humans.

## Introduction

CD4^+^ T cells arising from the thymus, expressing the FOXP3 transcription factor constitutively, denominated natural Treg cells, are instrumental for the maintenance of self‐tolerance.^1^ Treg cells can suppress the activation and effector function of other immune cells, including CD4 and CD8 T cells, B cells, NK cells, macrophages and dendritic cells.^2^ Congenital defect in Treg cells, for example because of a nonfunctional *foxp3* gene,^3^, ^4^, ^5^ or artificial ablation of Treg cells in adult animals^6^ leads to fatal systemic autoimmunity and immune dysregulation in the gut, indicating their crucial role in the prevention of autoimmune pathogenic events, lifelong. Those Treg cells are now designated thymic Tregs (tTregs) in the murine system.^7^

In addition to tTregs cells, peripheral CD4^+^T effector cells, that do not express FOXP3 constitutively, can acquire natural Treg cells function by upregulating FOXP3 upon activation in the presence of specific combinations of cytokines such as IL‐2 and TGF‐β^8^ or in the presence of small molecules such as retinoic acid.^9^

Treg cells induced in the periphery suppress immune responses as efficiently as tTregs cells. While tTreg cells are more prevalent in lymphoid organs and in peripheral blood and prevent immune responses towards self‐antigens, peripheral activation‐induced Tregs cells are more prevalent in mucosal tissues such as the gut^10^ in order to prevent local inflammation in the presence of exogenous antigens. Those peripherally induced Treg cells are henceforth denominated peripheral Treg cells (pTregs).

It is therefore well accepted in animals and humans that the pool of FOXP3^+^ Treg cells is heterogeneous, constituted of nTregs and pTregs, and it is possible to dissect the Treg cell pool based on several surface markers.

Treg subsets may have different functions or roles in the prevention of autoimmunity or other immune dysregulations. We discuss here how Treg cell subsets can be phenotypically differentiated in humans, how different they are in stability, epigenetics and function, and how Treg cell heterogeneity can affect the design of Treg biology‐based treatments.

## Heterogeneity in phenotype: human Treg cell subsets

**Figure 1:**
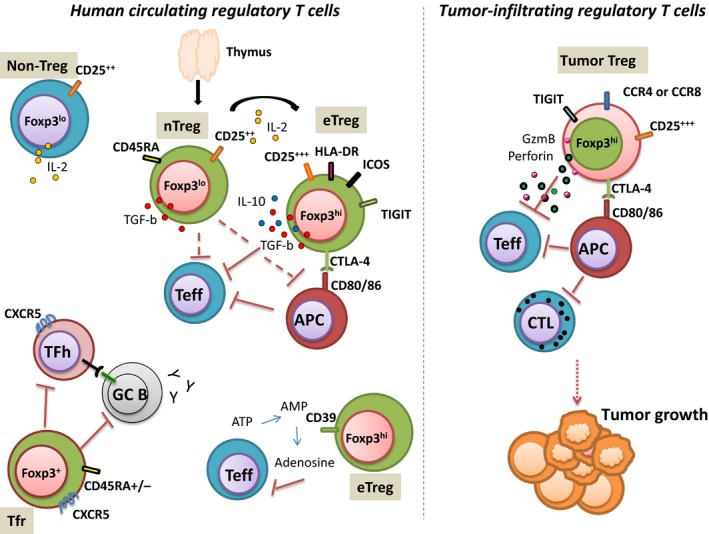
Heterogeneity in human Treg cell phenotype and function. Human circulating Treg cells are phenotypically and functionally heterogeneous. Different mechanism of suppression has been described in humans (contact‐dependent suppression, immunosuppressive cytokine secretion, cytolytic activity, IL‐2 adsorption). Some CD4^+^ T cells can express low levels of FOXP3 and secrete IL‐2. T follicular regulatory T cells that share phenotypic characteristics of TFH and of conventional Treg cells inhibit TFH and Germinal B cells. In tumor, infiltrating Treg cells differ phenotypically and functionally from circulating Treg. nTreg, naive regulatory T cells; eTreg, effector regulatory T cell; Teff, effector conventional T cell; APC, antigen‐presenting cell; DC, dendritic cell; CTL, cytotoxic T cell; ATP, adenosine triphosphate; AMP, adenosine monophosphate; GzmB, granzyme B; TFR, T follicular regulatory T cell; TFH, T follicular helper; GC B, germinal centre B cells.

While human regulatory T cells have been initially characterised phenotypically as a unique CD4^+^ T‐cell population with high expression of CD25 and then with low expression of CD127, it is now well accepted that the human Treg population is highly heterogeneous. For instance, mass cytometry analysis of human circulating Treg cells could easily identify more than 22 subsets.^11^ Because discrete differences in the expression of surface markers can lead to the definition of insignificant separate subsets, we only discuss here the key surface markers that enable the definition of distinct subsets in Treg cells in the periphery and in tumor tissues (Figure 1).

### Treg cell heterogeneity in the periphery

Three phenotypically and functionally distinct subsets can be developmentally defined in human CD4^+^T cells expressing the FOXP3 transcription factor: (1) CD45RA^+^ FOXP3^lo^ naïve or resting Treg (nTreg) derived from thymus, (2) CD45RA^−^ FOXP3^hi^ effector or activated Treg (eTreg) and (3) nonsuppressive CD4^+^ T cells with low expression of FOXP3. While nTreg and eTreg cells are highly suppressive and do not produce IL‐2, CD45RA^−^FOXP3^lo^ non‐Treg cells produce effector cytokine such as IL‐2, IL‐17 or IFN‐γ.^12^ The proportions of the three subpopulations can vary physiologically as eTreg cells number increases while nTreg cells number decreases with age. The prevalence of each Treg subsets can also vary during immune disease. For example, circulating eTreg cell number decreases during active systemic lupus erythematous while the proportion of eTreg cells increases in active sarcoidosis. The nTreg cells rapidly acquire the eTreg cell CD45RA^−^FOXP3^high^ phenotype when they have been activated *in vitro* or *in vivo* and it is well accepted that the eTreg cell compartment contains nTreg cells that have been activated. Our group has recently shown that sialyl Lewis x (CD15s) was highly expressed by eTreg cells in the periphery but not by FOXP3‐expressing CD45RA^−^ non‐Treg cells.^13^

Two other human Treg subsets can be defined in the thymus, in lymphoid organs and peripheral blood by the differential expression of ICOS (inducible T‐cell costimulator).^14^ The main mechanism of suppression of ICOS^+^ Treg is based on the secretion of IL‐10, whereas ICOS^−^ Treg cells produce membrane and secretory TGFb. ICOS^+^ FOXP3^+^ Treg grossly corresponds to eTreg whereas ICOS^−^ FOXP3^+^ Treg cells correspond with a naive phenotype.

In mice, the Helios transcription factor has been identified as a marker enabling the discrimination between thymic Treg cells (Helios^+^) and peripheral Treg cells (Helios^−^). Surface markers enabling the isolation of Helios^+^ Treg cells in humans have been described, that is, TIGIT and FCRL3.^15^ Nevertheless, FCRL3 has also been described as a Treg cell marker for poor proliferative response in the presence of IL‐2 and reduced suppressive capacities.^16^ Therefore, it is still controversial in humans whether the Helios maker is as discriminative as observed in mice.^17^ Neuropilin‐1 has also been described as a surface marker enabling the discrimination between murine tTregs and pTregs^18^, ^19^ but such finding could not be observed in humans.^20^, ^21^ It is therefore still uncertain how to dissect tTregs from pTreg cells in humans.

Nonetheless, it is well accepted that most if not all naïve Treg cells are thymic Treg cells because Treg cells emigrating from the thymus bearing the CD31 marker reside in the nTreg cell population.^12^, ^22^ Naïve Treg cells are also heterogeneous since they can be separated into recent thymic emigrants (CD31^+^) and other naïve Treg cells (CD31^−^) but it has not been investigated in depth whether the CD31^−^ compartment of nTreg cells is developmentally or functionally different from their CD31^+^ counterparts.

The CD45RA^−^ eTreg cell subset is also highly heterogeneous. Several surface markers such as HLA‐DR,^23^ TIGIT (T‐cell immunoreceptor with Ig and ITIM domain), GITR, LAG3 or CD39^24^, ^25^, ^26^, ^27^, ^28^ have been associated with stronger suppressive activity within the effector Treg cell subset.

### Treg cell heterogeneity in tumors

Treg cells can prevent immune response against tumor and promote tumor growth. The study of numerous tumor types (breast, lung, colon, etc.) has shown a consistent FOXP3^+^ Treg cell infiltration within the tumor and in the tumor microenvironment.^29^ In most tumor types, the prevalence of infiltrating Treg cells is correlated with the progression of the disease and with poor survival. Therefore, Treg cell removal in patients with cancer can be considered a promising therapeutic strategy to enhance immune responses against tumors. Heterogeneity is also observed in Treg cells infiltrating the tumors as they are phenotypically different from circulating Treg cells and they can differ according to the type of tumors. For example, tumor‐infiltrating Treg cells overexpress some surface molecules such as LAG3, TIGIT, CTLA4 or ICOS in both primary tumor or in metastatics when compared to circulating Treg cells.^30^ It should be noted that in human breast and colon cancer, infiltration by CCR8^+^ Treg and CCR4^+^ Treg cells is correlated with poorer prognosis, respectively.^31^, ^32^ Therefore, molecular targets to neutralise in Treg cells during tumor immunotherapy may differ given the type of tumors.

### Heterogeneity in the expression of effector transcription factors

Treg cells can also be separated based on their expression of T effector transcription factors such as those involved in the polarisation of effector T helper cells. T‐bet and RORC transcription factors are instrumental in TH1 and TH17 polarisation, respectively. Though it has been long believed that the expression of FOXP3 and of the other TH polarisation transcription factors were mutually exclusive, it has been proven, on the contrary, that the co‐expression by Treg cells of effector transcription factors T‐bet and RORC was indispensable for Treg cells to suppress TH1 and TH17 cells, respectively, in mice.^33^, ^34^ T‐bet‐expressing Treg cells and RORC‐expressing Treg cells share surface molecules with their effector T‐cell counterparts such as chemokine receptors that allow Treg cells to migrate where the effector cells are localised to suppress their function. Those TH1‐like Treg and TH‐17‐like Treg cells have also been described in humans.^35^ TH‐1 like Treg cells express CXCR3 while TH‐17 like Treg cells bear TH‐17 chemokine receptor CCR6 and membrane molecules such as CD161.^36^, ^37^

Regarding the TH2 transcription factor GATA‐3, studies in mice have indicated that some Treg cells could co‐express GATA‐3 and FOXP3. In the tissues, GATA‐3^+^ Treg cells can promote tissue regeneration especially in the muscles.^38^, ^39^ Such tissue Treg cells bear the IL‐33 receptor ST2. It is still unclear whether circulating GATA‐3^+^ Treg cells and tissue GATA‐3^+^‐Treg cells are developmentally related. It is also unclear whether GATA‐3‐expressing Treg cells can maintain their suppressive capacities or rather acquire effector TH2 cell functionality in the periphery in humans.

### Heterogeneity in T follicular regulatory cells

In addition to classical T helper cells that can be separated into effector T cells and regulatory T cells, T follicular helper cells (TFH), a subset of CD4^+^ T cells that are involved in the maturation of B cell in germinal centre (GC), can also be separated into effector T follicular cells and regulatory T follicular cells (TFR) based on their differential expression of FOXP3. FOXP3‐expressing TFR cells play critical roles in the control of TFH cells and GC formation, and they can be detected in the periphery with their expression of CXCR5. Human TFR cells display a particularly weak expression of CD25 in humans.^40^ Recent findings indicate that CD45RA expression can, as it is observed with conventional Treg cells, distinguish two subsets of TFR cells. However, CD45RA does not seem to be, in TFR cells, a marker indicating a thymic origin since CD45RA^+^ TFR cells are absent from the thymus or cord blood.^41^ Therefore, the significance of the expression of CD45RA on human TFR cells in the periphery remains unclear.

## Treg cell heterogeneity in stability and epigenetics

**Figure 2:**
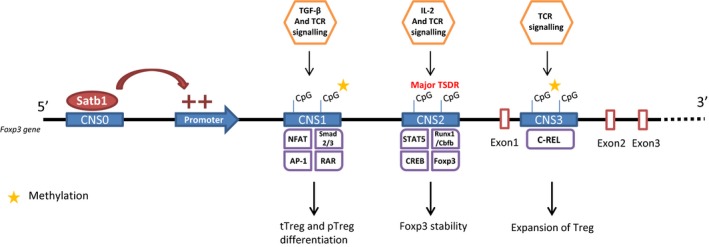
Treg epigenetics: role of conserved noncoding sequences on Treg cell lineage stability. *Foxp3* gene CpG demethylation is required for Treg suppressive function and lineage stability. The methylation status of four conserved noncoding sequences (CNS0, CNS1, CNS2, and CNS3) contributes, respectively, to FOXP3 expression, tTreg and pTreg differentiation, stability of FOXP3 expression and Treg expansion. Satb1 ligation on CNS0 acts as a ‘super‐enhancer’ for *foxp3* expression initiation. Illustration based on Kanamori *et al*.^44^ and Iizuka‐Koga *et al*.'s^45^ papers. Satb1, Special AT‐rich sequence binding protein 1; NFAT, nuclear factor of activated T cells; AP‐1, activator protein 1; RAR, retinoic acid receptor; STAT5, signal transducer and activator of transcription 5; Runx1, runt‐related transcription factor 1; Cbfb, core binding factor beta, CREB, cAMP response element binding protein, Foxp3, forkhead box P3; TSDR, Treg‐specific demethylated region; TCR, T‐cell receptor; TGFb, transforming growth factor beta, IL‐2, interleukin‐2; tTreg, thymic regulatory T cell; pTreg, peripheral regulatory T cell.

Treg cell lineage‐specific transcriptional factor FOXP3 is considered as a master regulator of Treg cell function. Epigenetic gene modifications, such as DNA methylation, DNA acetylation and histone modification, are essential for regulating gene expression required for the stabilisation and fixation of a cell lineage.^42^ Indeed, Treg‐specific DNA hypomethylation pattern is required for the stability and development of tTreg cells.^43^ Hypomethylation is independent of FOXP3 expression but necessary for FOXP3^+^ Treg cells to acquire FOXP3‐independent gene expression, lineage stability and full suppressive activity. Recent studies have shown that methylation status of enhancers at the FOXP3 gene locus, designated as conserved noncoding sequences (CNSs) 1, 2 and 3, differentially contributes to tTreg and pTreg differentiation and stability (Figure 2).

### CNS1

The CNS1 enhancer contains binding sites for transcription factors, including Smads, NFAT, AP‐1 and retinoic acid receptor (RAR). TGF‐β signalling induces phosphorylated Smad2/3 to migrate in the nucleus and induce FOXP3 expression. TGF‐β1‐deficient mice and Smad2/3 double‐deficient mice exhibit relatively normal nTreg development in the thymus, but the peripheral Tregs are significantly reduced in number. Similarly, in mice, deletion of CNS1 in Treg cells abrogates differentiation of pTreg cell subset.^44^ Furthermore, Treg cell‐specific CNS1‐deletion in mice shows an accelerated mucosal Th2 inflammation, which indicates the importance of pTregs in the suppression of excessive immune responses in the mucosa.^45^

### CNS2

The CNS2 enhancer contains binding sites for other transcription factors, including Stat5, NFAT, Runx1/Cbfb, CREB and FOXP3 itself.^46^ The CNS2 locus is highly enriched with CpG sites, and the methylation status is a particularly important determinant for the activity of this enhancer. It has been shown that demethylation of the CpG islands in the CNS2 region of the *foxp3* locus is associated with stable expression of FOXP3 in tTregs.^47^ DNA demethylation at this locus is also observed in pTregs, although with slightly reduced levels compared with tT‐regs. *In vitro* induction of Treg cells (iTreg) in the presence of IL‐2 signals induces demethylation of CNS2, stabilized FOXP3 expression and stable Treg cell phenotype.^44^ However, this region is rarely demethylated in iTreg cells in the absence of IL‐2 signalling, which is why FOXP3 expression in this cell subset is highly unstable. Deletion of CNS2 region in mice revealed that this enhancer region is required for FOXP3 induction. Furthermore, CNS2 region is important for the maintenance of FOXP3 expression particularly under inflammatory conditions in which Tregs are exposed to inflammatory cytokines and stronger TCR stimulation.^44^ Additionally, full‐methylation of this locus is reported to be important for abnormal FOXP3 induction in non‐Treg cells, including CD8 T cells and NK cells. Major Treg‐related demethylated regions (TSDRs) are also observed in promoter region of Ctla4, Il2ra (which encodes CD25), Ikzf4 (which encodes Eos) and Tnfrs18 (which encodes GITR). These results indicate that methylation status not only regulates FOXP3 expression but also maintains expression of FOXP3 regulated genes. Recently, it was reported that ten eleven translocation (Tet) family of demethylation factor plays an important role in CpG demethylation at CNS2 in Treg cells. T‐cell‐specific deletion of Tet1 and Tet2 in mice results in *foxp3* hypermethylation, impaired Treg cell differentiation and function, and autoimmune disease.^48^

In humans, CD45RA^+^ naïve Treg cells and FOXP3^high^ CD45RA^−^ effector Treg cells have been consistently described with fully demethylated CNS2/TSDR while FOXP3^low^ non‐Treg cells or *in vitro* induced FOXP3‐expressing CD4^+^ T cells have been described with only partially demethylated CNS2/TSDR.^12^, ^43^

### CNS3

The CNS3 enhancer contains a binding site for c‐Rel, and in CNS3 enhancer‐deleted mice, Treg development is impaired. CNS3‐deficient mice show increased numbers of activated effector T cells and elevated IL‐13 and IFN‐γ production by T cells in the lungs and increased titres of circulating autoantibodies against several self‐antigens. It is reported that CNS3 is an intronic *foxp3* regulatory element, and demethylation of this region increases the probability of FOXP3 induction in response to TCR stimulation, particularly within a lower range of signal strength. Epigenetic changes in CNS3 therefore regulate the expansion of Treg cell repertoire to control self‐reactive T cells effectively.^49^

### Super‐enhancer

Kitagawa *et al*.^50^ assessed epigenetic and transcriptional regulation in peripheral Treg cells to identify a class of enhancers, called super‐enhancers, regulating several genes that define Treg cell identity, including *foxp3*,* Ctla4* and *Il2ra*. Using extensive genomic and epigenetic analysis, they confirmed that the three conserved noncoding sequence elements (CNS1–CNS3) at the *foxp3* locus were associated with enhancer activity that is important for the differentiation of Treg cells and the stability of *foxp3* expression. They also identified another region, approximately 8‐kb upstream of the transcriptional start site, called CNS0, to be crucial for the expression of FOXP3 in Treg cells. This region binds to Satb1, a global genome organiser that induces both transcriptional regulation and epigenetic regulation via the formation of a novel nuclear architecture. It is proposed that Satb1 binds to CNS0 and then alters the chromatin accessibility for histone modifications. Thus, Satb1 functions as a pioneering element that is required for subsequent activities of the other CNS elements that lead to initiation, commitment and stability of FOXP3 expression.^50^

How Satb1 is differentially expressed in human Treg cell subsets is not known yet.

Treg cells can be defined not only with the expression of FOXP3 and surface markers but also based on a specific epigenetic landscape that is necessary for the stability of Treg cells. Because some FOXP3‐expressing CD4^+^T cells such as FOXP3^low^ CD45RA^−^CD4^+^ T cells do not harbour the epigenetic profile of naïve Treg cells or of effector Treg cells,^12^, ^43^ it is not surprising that these cells are unstable in expressing FOXP3 or in maintaining Treg cells properties (i.e. absence of production of IL‐2 or suppressive function).

## Heterogeneity in Treg mechanisms of suppression

Treg cells utilize a wide range of suppressive mechanisms, including IL‐2 depletion, physical deterrence of the access of responder T cells to APCs, TGF‐β either membrane bound or secreted, immunosuppressive cytokines IL‐10 and IL‐35, production of FGL2 after the ligation of TIGIT, CD39/CD73 ectonuclease activity leading to the production of adenosine, cytotoxicity and CTLA‐4‐dependent mechanism.^51^

Based on numerous reports, it is now well established that all of the described mechanisms are not utilized simultaneously when Treg cells are suppressing immune responses. For instance, cytotoxicity by the production of perforin and granzyme B has been observed in some specific conditions in tumors.^52^ Treg cells can therefore be separated in functionally different subsets based on the mechanisms of suppression that are mainly utilized. For instance, Treg cells can be separated based on their production of IL‐10 or TGF‐B^14^ or their expression of CD39,^53^ or CTLA‐4^54^ (see Figure 1).

CTLA‐4 has been described as a key suppressive mechanism in Treg cells but not all Treg cells in human express CTLA‐4,^12^ and we discuss here Treg cell functional heterogeneity through CTLA‐4.

### CTLA‐4‐mediated *in vivo* suppression

Mice with deficient CTLA‐4 gene develop fatal systemic autoimmunity with lymphoproliferation.^55^ While effector T cells upregulate CTLA‐4 several days after activation, Treg cells in mice constitutively express CTLA‐4. Treg‐specific conditional gene deletion of CTLA‐4 in mice demonstrates a similar phenotype to that reported for *foxp3*‐deficient mice, that is mice suffer from hyper IgE, high concentrations of serum IgG and autoantibodies, and lymphoproliferative disease, in addition to fatal systemic autoimmunity.^54^ In humans, for decades, deficiency in CTLA‐4 was assumed to be fatal *in utero*. Some polymorphisms of CTLA‐4 gene had been described, usually associated with benign autoimmunity such as Grave's disease, but without defect in CTLA‐4 molecular function.^56^ However, in 2014, two independent studies gathered several families with multiple cases of germline heterozygous mutations with clinical severe autoimmunity including enteropathy, interstitial lung disease, kidney and/or liver infiltration, encephalitis, psoriatic lesions and autoimmune cytopenia.^57^, ^58^ Of note, Treg cell expression of CTLA‐4 was weaker in patients when compared to healthy donors especially after activation.

While in mice, all Treg cells express CTLA‐4,^59^ naïve Treg cells do not express CTLA‐4 in humans. Naïve Treg cells upregulate CTLA‐4 upon activation as effector CD4^+^ T cells do. This may indicate that in humans, on one hand, a subset of Treg cells that express CTLA‐4 is required to prevent a defined spectrum of autoimmune diseases that are seen in patients with CTLA‐4 haploinsufficiency. On the other hand, some other subsets of Treg cells that do not express CTLA‐4 but utilise other mechanisms of suppression may be required to prevent other autoimmune diseases. A long asymptomatic period is observed before the onset of the clinical autoimmune diseases in CTLA‐4 haploinsufficiency, we can hypothesise that CTLA‐4 deficiency can be compensated by other Treg mechanisms of suppression for years but eventually leads to autoimmune diseases because Treg cell mechanisms of suppression are not redundant.

### CTLA‐4 blockade in tumor immunotherapy

An important role for CTLA‐4 modulation in human disease is further validated in clinical studies. CTLA‐4 antibody treatment blocks immunosuppressive functions of Treg cells and induces negative costimulatory signals in T effector cells. There are currently 11 programs with anti‐CTLA‐4 therapies: ipilimumab, Bristol‐Myers Squibb launched, tremelimumab, medimmune in phase 3 and MK‐1308, Mercks & Co, in phase 1 and 7 in the preclinical stages from CytoMX Therapeutics, Bristol‐Myers Squibb, Abzena (PolyTherics), BioAtla, Aduro BioTech, Anaeropharma Science (anti‐CTLA‐4 alone and in combination with anti‐PD1), Tikcro Technologies, Aida Pharmaceuticals (Citeline, August 2017).

Indeed, ipilimumab showed tumor regression with a prolonged response.^60^ In the phase 3 study on survival rate with patients with metastatic melanoma (stage III and IV), Hodi *et al*. (2010) demonstrated overall survival rate. However, some patients (10–15%) had immune‐related adverse effects.^61^ Seven of 14 deaths were related to immune‐related adverse effects, affecting mainly the skin, the pituitary gland and the gastrointestinal tract. This indicates that *in vivo* suppression mediated by Treg cells through CTLA‐4 participates in the prevention of antitumor immune responses and, as described above with CTLA‐4 haploinsufficiency, of autoimmunity and/or immune dysregulation in the skin, the gut and in some endocrine organs.

## Treg cell heterogeneity and the design of Treg biology‐based treatments

### Low‐dose IL‐2 and Treg cell *in vivo* expansion

Initial testing of the potential use of Treg cells in the clinic was done in graft‐versus‐host disease following allogeneic hematopoietic stem cell transplantation.^62^ These studies were followed by several reports evaluating Treg cell‐based therapies in autoimmune and transplantation patients. These studies established for safety profile for the use of Treg cells in the clinic showed mixed results.

However, it was also identified in these studies that a subset of Treg cells is unstable, with reduced Treg‐specific determining regions (TSDR/CNS2) demethylation and potential production of pathogenic cytokines, such as IFN‐g. As discussed above, the stability of FOXP3 protein in human CD4^+^ T cells after T‐cell activation depends on DNA demethylation of CNS intronic regions of the *foxp3* gene. Thus, new methodologies are necessary to stabilise the Treg cell phenotype. Recent studies on expansion protocols evaluating low‐dose IL‐2 together with rapamycin showed improved stability of the circulating Treg cells of T1DM patients, at least 1 year post‐treatment.^63^ Although similar combination in preclinical models resulted in prevention of both spontaneous diabetes and recurrent diabetes after islet transplant,^64^ in this phase 1 clinical trial, the combination of rapamycin and low‐dose IL‐2 therapy was not convincing as, despite a rise in circulating Tregs cells, a decline in C‐peptide values was observed. On the negative side, a significant increase in effector T cells and in NK cells was observed, promoting a proinflammatory environment causing transient β‐cell dysfunction. These results highlight the difficulties in translating experimental findings to the clinics and emphasise the importance of broadly interrogating the immune system to evaluate the effects of therapy, especially protocols to dissociate Treg cell expansion from effector responses.

### Utilising CTLA‐4‐mediated Treg cell suppressive function in autoimmunity

The cytotoxic T lymphocyte‐associated antigen 4 Ig fusion protein (CTLA‐4 Ig fusion protein) has been approved in the rheumatoid arthritis disease with Abatacept (Bristol‐Myers Squibb) and in transplant rejection with Belatacept (Bristol‐Myers Squibb). The difference between these two Ig fusion proteins is that Belatacept, a derivative of the CTLA‐4 Ig, contains two amino acid substitutions.^65^ CTLA4‐Ig mimics the function of cell surface expressed CTLA‐4 on Treg cells and on activated effector T cells by binding to CD80 and CD86 on APC, resulting in inhibition of T effector function.

The first CTLA‐4 Ig fusion protein to demonstrate efficacy in rheumatoid arthritis over disease‐modifying antirheumatic drugs with low incidence rate for serious adverse effects like serious infection was abatacept.^66^ The total infection rate was increased (37.6% compared with 32.3% for placebo) but no increase in serious infections, or in discontinuations due to infection was reported in the treated group compared to placebo.^67^ Of interest to notice was with patients with comorbidity. In cases of patients with COPD for example, they exacerbated more frequently on abatacept treatment compared to placebo, due to an increase in respiratory and infection‐related events.^67^ There is a low incidence of immunogenicity reported with abatacept both in the i.v and in the s.c administration, with s.c administration having similar safety and efficacy profile compared to i.v.^68^

Belatacept was investigated in two‐phase 3 clinical trials in patients receiving kidney transplant: the BENEFIT and BENEFIT‐EXT studies. In the BENEFIT study, two dose regimens of belatacept were investigated. Vincenti *et al*. (2010) demonstrated that the renal function measured in the composite renal impairment endpoint and by the mGFR was significantly increased with belatacept‐treated patients compared with cyclosporine‐treated patients. No differences were noticeable within the two dosing regimens in term of efficacy or safety. However, the patients under belatacept had a higher rate of acute rejection episodes and 1‐year survival rate was similar in the two different drug‐treated groups.^69^ The acute rejection incidence was lower in lower regimen group. Furthermore, the donor‐specific antibodies, risk of death or graft loss investigated during the 7‐year follow‐up study was significantly lower in the belatacept‐treated patients than in the cyclosporine‐treated patients from BENEFIT study.^69^

The BENEFIT‐EXT study investigated the effect of belatacept treatment in patients receiving a kidney transplant from an extended criteria donor with at least two other risk factors (cerebrovascular accident, hypertension or serum creatinine >1.5 mg dl^−1^); or an anticipated cold ischaemia time of ≥24 h; or donation after cardiac death.^70^ In this group, belatacept‐treated patients showed better renal function and an improved cardiovascular/metabolic profile versus cyclosporine‐treated patients. However, patient/graft survival and acute rejection were similar in both groups. It is worth noting there was an increase in post‐transplant lymphoproliferative disorder (PTLD) cases with belatacept with three of the five PTLD patients having Epstein–Barr virus negative serology.

In the light of the recent results of these clinical trials, there is currently a need to understand the patient's disease mechanism of action in order to find more selective targets. For instance, the use of CTLA‐4‐Ig does not seem to recapitulate the whole spectrum of Treg‐related suppression. Other molecules might be used to mimic other Treg mechanisms of suppression in order to obtain optimised control of immune responses in transplantation and autoimmune diseases.

## Conclusion

Human Treg cells are heterogeneous in phenotype, function and epigenetics. Old human studies have focused on Treg cells as a sole homogeneous population.^71^ New insights in the definition of Treg cell subsets should enhance our knowledge on the role of each subset in health and diseases in order to manipulate certain subsets of Treg cells, not all, in order to provide better Treg biology‐based innovative therapeutic strategy to restore immune homeostasis or to promote tolerance.

## Conflict of interest

The authors declare no conflicts of interest.
